# Longitudinal wastewater-based surveillance of SARS-CoV-2 during 2023 in Ethiopia

**DOI:** 10.3389/fpubh.2024.1394798

**Published:** 2024-10-07

**Authors:** Daniel Abera Dinssa, Gebremedhin Gebremicael, Yohannes Mengistu, Noah C. Hull, Dinknesh Chalchisa, Girma Berhanu, Atsbeha Gebreegziabxier, Ashley Norberg, Sarah Snyder, Sarah Wright, Waktole Gobena, Adugna Abera, Yohannes Belay, Dawit Chala, Melaku Gizaw, Mesay Getachew, Kirubel Tesfaye, Mesfin Tefera, Mahlet Belachew, Tegegne Mulu, Solomon Ali, Abebaw Kebede, Daniel Melese, Saro Abdella, Tobias F. Rinke de Wit, Yenew Kebede, Mesay Hailu, Dawit Wolday, Masresha Tessema, Getachew Tollera

**Affiliations:** ^1^Ethiopian Public Health Institute (EPHI), Addis Ababa, Ethiopia; ^2^Global Health, The Association of Public Health Laboratories (APHL), Addis Ababa, Ethiopia; ^3^Global Health and Environmental Health, The APHL, Bethesda, MD, United States; ^4^Environmental Health, The APHL, Bethesda, MD, United States; ^5^Department of Microbiology, Immunology and Parasitology, St. Paul’s Hospital Millennium Medical College, Addis Ababa, Ethiopia; ^6^Africa Centres for Disease Control and Prevention (Africa CDC), Surveillance and Disease Intelligence Division, Addis Ababa, Ethiopia; ^7^Amsterdam Institute of Global Health and Development, Department of Global Health, Amsterdam University Medical Center, Amsterdam, Netherlands; ^8^Department of Biochemistry and Biomedical Sciences, Michael G. DeGroote Institute for Infectious Diseases Research and McMaster Immunology Research Center, Faculty of Health Sciences, McMaster University, Hamilton, ON, Canada

**Keywords:** COVID-19, SARS-CoV-2, qRT-qPCR, wastewater treatment plants, wastewater-based epidemiology

## Abstract

**Introduction:**

Although wastewater-based epidemiology (WBE) successfully functioned as a tool for monitoring the coronavirus disease 2019 (COVID-19) pandemic globally, relatively little is known about its utility in low-income countries. This study aimed to quantify severe acute respiratory syndrome coronavirus 2 (SARS-CoV-2) RNA in wastewater, estimate the number of infected individuals in the catchment areas, and correlate the results with the clinically reported COVID-19 cases in Addis Ababa, Ethiopia.

**Methods:**

A total of 323 influent and 33 effluent wastewater samples were collected from three Wastewater Treatment Plants (WWTPs) using a 24-h composite Moore swab sampling method from February to November 2023. The virus was captured using Ceres Nanotrap® Enhancement Reagent 2 and Nanotrap® Microbiome A Particles, and then nucleic acids were extracted using the Qiagen QIAamp Viral RNA Mini Kit. The ThermoFisher TaqPath™ COVID-19 kit was applied to perform real-time reverse transcriptase polymerase chain reaction (qRT-PCR) to quantify the SARS-CoV-2 RNA. Wastewater viral concentrations were normalized using flow rate and number of people served. In the sampling period, spearman correlation was used to compare the SARS-CoV-2 target gene concentration to the reported COVID-19 cases. The numbers of infected individuals under each treatment plant were calculated considering the target genes’ concentration, the flow rate of treatment plants, a gram of feces per person-day, and RNA copies per gram of feces.

**Results:**

SARS-CoV-2 was detected in 94% of untreated wastewater samples. All effluent wastewater samples (*n* = 22) from the upflow anaerobic sludge blanket (UASB) reactor and membrane bioreactor (MBR) technology were SARS-COV-2 RNA negative. In contrast, two out of 11 effluents from Waste Stabilization Pond were found positive. Positive correlations were observed between the weekly average SARS-CoV-2 concentration and the cumulative weekly reported COVID-19 cases in Addis Ababa. The estimated number of infected people in the Kality Treatment catchment area was 330 times the number of COVID-19 cases reported during the study period in Addis Ababa.

**Discussion:**

This study revealed that SARS-CoV-2 was circulating in the community and confirmed previous reports of more asymptomatic COVID-19 cases in Ethiopia. Additionally, this study provides further evidence of the importance of wastewater-based surveillance in general to monitor infectious diseases in low-income settings.

**Conclusion:**

Wastewater-based surveillance of SARS-CoV-2 can be a useful method for tracking the increment of COVID-19 cases before it spreads widely throughout the community.

## Graphical abstract

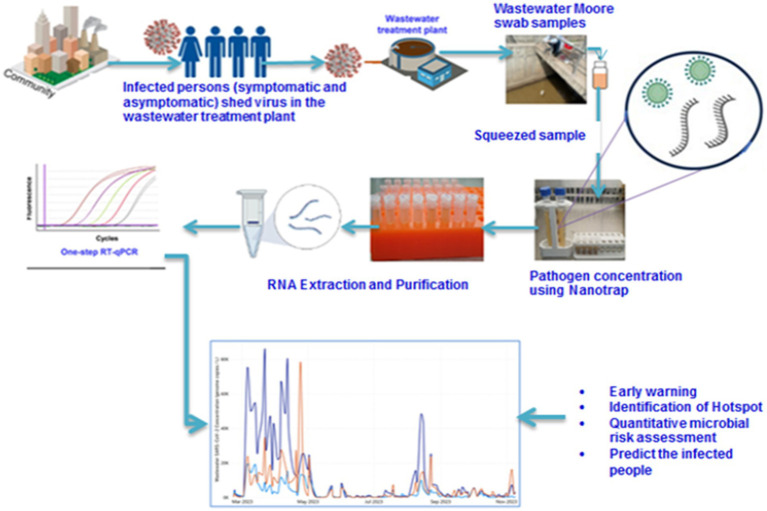

## Background

Economic stability and human health are considerably affected by infectious diseases as they cause one-fourth of the mortalities around the world (1). The recent outbreak of COVID-19 caused by severe acute respiratory syndrome coronavirus 2 (SARS-CoV-2) escalated into a global pandemic since it first appeared in Wuhan, China, in December 2019 (2). In January 2020, it led to a declaration of a Public Health Emergency of International Concern by the World Health Organization (WHO) (3). Since then, SARS-CoV-2 has been responsible for more than 773 million confirmed cases and around 7 million deaths worldwide as of December 2023 (4). In this regard, Africa reported only around 1.2% of confirmed cases and 2.5% of deaths. The first COVID-19 case in the African continent was reported from Egypt on the 14th of February 2020 (5). On February 25, Nigeria became the second country to report a first case, and on February 27, Algeria became the third country to do so (6). The first cases in other African countries, including Ethiopia, were only detected in March 2020 (7). Most index cases originated in Europe, where the epidemic’s epicenter had moved by March 13. As a result, the pandemic spread quickly to Africa (8). Consequently, this led to long-lasting collateral damage on the continent from interruptions in the initiatives for TB, HIV/AIDS, malaria, and vaccine-preventable illnesses (9). Ethiopia reported around 5 and 4.3% of the African total confirmed cases and deaths, respectively (4).

Surveillance focused on clinical and laboratory testing which has drawbacks such as excessive costs, failure to detect asymptomatic patients, and underestimating of infection prevalence (10). Current data suggest that worldwide 35–45% of all SARS-CoV-2 infections account for asymptomatic infected persons (11–13). However, the percentage of asymptomatic cases in Africa and Ethiopia is 67 and 74%, respectively (14–16). Recent study findings in Ethiopia indicated that high asymptomatic cases are associated with persistently activated immune system (17, 18). This will affect the clinical COVID-19 case detection and reporting as testing of samples was prompted mainly by symptoms (19). Hence, the community may not be prepared in terms of infection prevention and control, and management of COVID-19 infection (20).

SARS-CoV-2 RNA can be detected in feces and urine from asymptomatic and symptomatic individuals. Fecal shedding can persist for several weeks, typically longer than positivity in oropharyngeal swabs (21, 22). The extended presence of viral RNA in feces and fecal viral RNA shedding with gastrointestinal (GI) symptoms implies that SARS-CoV-2 infects the GI tract (23–25). Anyhow, virus shedding in the feces of symptomatic and asymptomatic infected individuals enables the detection of viral RNA in influent sewage or wastewater (26, 27). Wastewater-based epidemiology (WBE) for COVID-19 surveillance can be used as an alternative for early warning of COVID-19 outbreaks or as a control mechanism for potential virus transmission independent of individual healthcare-seeking behaviors. In addition, WBE can be scaled relatively easily, is less expensive than human subject testing, and, if collected at strategic points, can represent local populations (28, 29). Monitoring SARS-CoV-2 circulation in the community will remain important for reinforcing preparedness and identifying hotspots for further classical surveillance interventions, particularly in regions with inadequate health system infrastructure, human resources, and testing capacity.

Previously, numerous human infectious illnesses (such as polio and typhoid) have been the focus of research in this WBE (30, 31). In high-income countries, wastewater-based surveillance is well utilized for the monitoring of SARS-CoV-2 (32). However, few African countries have conducted wastewater-based SARS-CoV-2 surveillance (33–35). This may be partly attributable to low sewage coverage with deficient testing coverage, which limits COVID-19 surveillance through sewage monitoring (36). In Ethiopia, there is only one study in wastewater-based SARS-CoV-2 using a small sample size, and it is focused only on the qualitative test (37). This study aimed to quantify SARS-CoV-2 RNA in wastewater, estimate the number of infected individuals in the catchment area, and correlate results with clinically reported COVID-19 cases in Addis Ababa, Ethiopia.

## Materials and methods

### Study setting and sampling sites

Addis Ababa is Ethiopia’s capital city, with an estimated 5,460,591 population in 2023 (38). Administratively, it is divided into 11 subcities. Based on the information obtained from Addis Ababa Water and Sewerage Authority (AAWSA), the wastewater treatment capacity in Addis Ababa is nearly 86%, out of which 34% are currently connected to sewer lines, and 52% rely on vacuum trucks, the remaining could be considered as illegal connection or disposal. Currently, Addis Ababa city has 4 centralized and 35 decentralized wastewater treatment plants (WWTPs). The centralized WWTPs are Kality, Kality old, Kotebe old, and Chefe (unpublished Strategic Environmental and Social Assessment [SESA] of Addis Ababa City Sanitation Master Plan, 2024).

**Figure 1:**
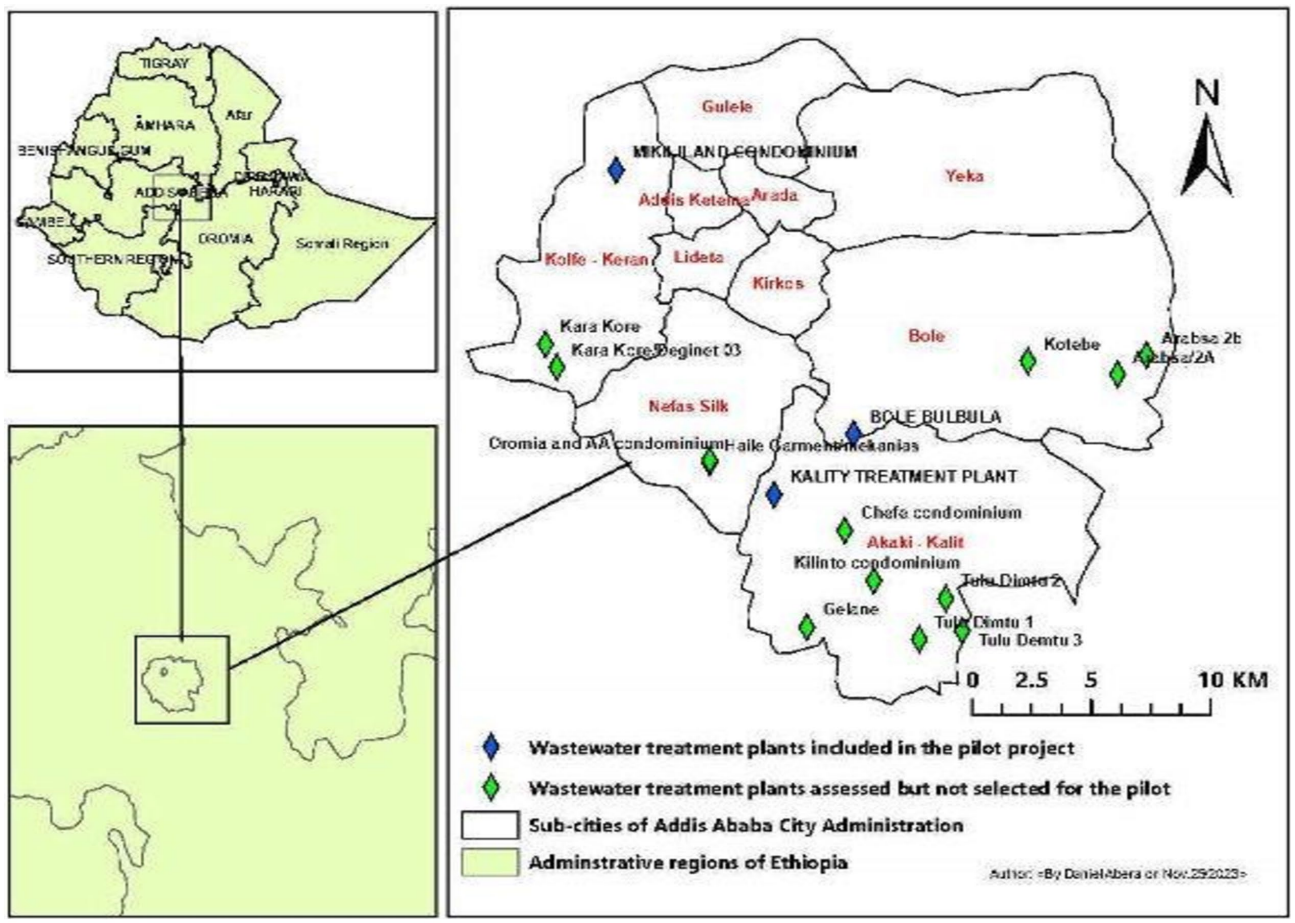
Sites of wastewater treatment plants. Map of wastewater treatment plant units in Addis Ababa, where all sites with diamonds were preliminary assessed. Blue diamonds represent selected sites, whereas green diamonds represent unselected sites due to different criteria.

Influent wastewater samples were collected 3 times a week from three sampling sites (Kality, Bulbula, and Mikililand) using the Moore swab method (39) (Figure 1). Kality treatment plant (KTP) is the oldest centralized system, mainly serving residents in the central, southern, and eastern parts of the city (40) with an estimated population coverage of nearly 2,000,000 (unpublished data from AAWSA). The upflow anaerobic sludge blanket reactor (UASB) technology is applied at this site. A membrane Bioreactor (MBR) wastewater treatment technology, which combines a biological-activated sludge process and membrane filtration domestic wastewater treatment, is used at the Bulbula wastewater treatment site (41). The third wastewater treatment plant included in this study was Mikililand Waste Stabilization Pond (WSP). Mikililand WSP systems comprise 7 series of different types of ponds (42). It is situated in the northwestern part of the capital city. Technical details of the wastewater treatment process at the three wastewater treatment plants are presented in Table 1.

**Table 1:** Description of the selected WWTPs.

	WWTP name	Sub-city/location	Design capacity m^3^ per day	Average daily flow rate in m^3^ per day	Served population	Type of treatment technology in place
1	Kality treatment plant (KTP)	Akaki Kality Sub-City/ Southern Addis Ababa	100,000^a^	65,000	2,000,000	USAB
2	Bulbula Treatment Plant	Bole Sub-City/ Central Addis Ababa	20,000^b^	325	34,000	MBR
3	Mikililand Wastewater Stabilization Pond (WSP)	Kolfe-Keranio Sub-City/ Northwest Addis Ababa	3,000^c^	NA	24,000	WSP

Description of the selected WWTPs for this surveillance of SARS-CoV-2 in Addis Ababa, Ethiopia. ^a^Information was collected from the AAWSA authority. ^b,c^Information was from the literature that is cited (37) and (42). NA = Not available (we did not know the average flow rate due to lack of measuring instruments at the WSP).

### Study design and sample collection

A longitudinal study design was conducted between February and November 2023 at three wastewater treatment plants in Addis Ababa. The Moore swab, or cotton gauze of size (120 × 15 cm), was folded to achieve an 8-ply pad and tied with a string that was long enough to immerse the swab into the influent discharge (39, 43). The prepared Moore swab was then autoclaved and sealed in Ziploc® bag. The string was attached to a solid structure and fully submersed into the wastewater. On all three wastewater collecting sites, the swab installation period was between 9:30 a.m. and 11:30 a.m. on Sundays, Tuesdays, and Wednesdays of each week. Following a 24-h period for the installation of the swab, the wastewater from the submersion was collected and placed in a Ziploc® bag. Finally, the exterior of Ziploc® bag was decontaminated with 70% ethanol and then transported using an ice-cold box to the Ethiopian Public Health Institute’s laboratory. Accordingly, 323 influent and 33 effluent wastewater samples were collected. The influent samples were composed of 110 from Kality, 108 from Bulbula, and 105 from Mikililand treatment plants Whereas, 11 effluent samples were collected from each of the three treatment plants with the same installation time of influent samples in October 2023 and November 2023.

Furthermore, to evaluate the effectiveness of the Moore swab sampling method in capturing virus particles from wastewater, influent samples were collected in parallel, covering the same 24-h period for 3 weeks from February 2023 to March 2023 using an on-site autosampler placed at KTP (*n* = 8).

### RNA capture and extraction

Each Moore swab was squeezed of all liquid into a sterile container from which a 10 ml wastewater aliquot was taken using a 15 ml tube for RNA capture and extraction. For RNA concentration, 100 μl of Nanotrap® Enhancement Reagent 2 (ER2; SKU# 10112, Ceres Nanoscience, Inc., Manassas, VA) and 150 μl of Nanotrap® Microbiome A Particles (SKU#44202, Ceres Nanosciences, Inc., Manassas, VA, USA) were added into the 15-ml tube containing 10-ml wastewater and mixed well. After samples were incubated at room temperature for 10 min, Nanotrap® Microbiome A Particles pellet was separated using a DynaMag™-15 Magnet (Thermo Fisher Scientific, Waltham, MA, USA). After washing the pellet, 150 μl of 1× phosphate-buffered saline (PBS) for suspension and 5 μl of MS2 phage control were added to each pellet, and negative control (RNAse free water); then RNA extraction was executed using QIAamp Viral RNA Mini Kit (QIAGEN, Hilden, Germany) following the manufacturer’s instruction (44). MS2 spike-in to each sample can minimize false negatives. Briefly, 560 μl QIAGEN Virus Lysis Buffer was added to PBS suspended pellet to lyse the cells. Following a 10-min incubation at room temperature of the solution, the Nanotrap® Microbiome A Particles and the lysate solution sample were separated using the DynaMag™-2 magnet (Thermo Fisher Scientific, Waltham, MA, USA). The lysate supernatants were collected in a new 1.5-ml microcentrifuge tube, and the pellet was discarded. For high nucleic acid concentration, 560 μl of 100% ethanol was added to the concentrate, and the lysate was added to the QIAamp Mini column. After washing using wash buffer, the QIAamp Mini column was placed in a clean 1.5-ml microcentrifuge tube, elution was performed using 80-μl Buffer AVE, and the eluted viral RNA was stored at −80°C.

#### Real-time reverse transcriptase polymerase chain reaction (qRT-PCR)

The TaqPath™ COVID-19 control was used as a quantification standard RNA control (1 × 10^4^ copies/μl stock). A 10^4^ copies/μl was diluted to 2 × 10^3^ copies/μl using dilution buffer and then used as stock. The stock solution was then serially diluted 5-fold in low-binding 1.5-ml tubes. The limit of detection of the TaqPath™ COVID-19 is 10 genomic copy equivalents (GCE)/reaction (45), but we did not do the limit of detection in our setting.

TaqPath™ COVID-19 qRT-PCR reaction master mix was prepared according to the manufacturer’s instructions (45). A total of 15-μl master mix was added to each well of the plate. Approximately 10 μl of extracted nucleic acid, quantification standard RNA, and nuclease-free water for no template control (NTC) were added to the assay wells containing the master mix. In the Plate Setup window of QuantStudio™ 5 (Thermo Fisher Scientific, Waltham, MA), FAM, VIC, ABY, and JUN dyes were used as reporter dyes for the viral targets of the primers and probes: ORF1ab, Nucleocapsid (N) gene, Spike (S) gene, and MS2 phage control, respectively (45). Thermal cycling conditions included 2-min of uracil-N-glycosylase (UNG) incubation at 25°C, 10-minu of reverse transcription at 53°C, 2-min at 95°C for reverse transcription deactivation, and initial activation of Speed Star HS DNA polymerase, followed by 40 cycles of 3 s denaturation at 95°C and 30 s annealing/extension at 60°C. All samples with cycle threshold (Ct) values of ORF1ab, N gene, and S gene <37; MS2 < 32 were considered positive according to the manufacturer (45).

#### Determination of viral concentration in wastewater

The PCR test results were interpreted as follows: when any two or more of the viral targets were reported, the sample was considered positive for SARS-CoV-2; when only one viral target was detected within repeated tests, the result was considered inconclusive; whereas when all the viral targets were not detected but the internal control (MS2) detected, the sample was considered as negative for SARS-CoV-2. Preliminary reverse transcriptase qPCR data analysis and quality control were performed using the QuantStudio Flex 5 reverse transcriptase qPCR software v1.5.1 (Applied Biosystems, Inc., USA). Viral concentrations were expressed as genome copies of RNA extract per liter (gc/L). Using Excel and the following formula, viral concentrations (gc/L) in the concentrated samples were determined:


$$\begin{array}{l} \mathrm{Concentration of viral genome in wastewater}\;\left(\frac{\mathrm{copies}}{L}\right)=\\ \frac{\mathrm{Copies in}\;\mathrm{RT}-\mathrm{qPCR reaction}\left(\mathrm{copies}\right)\;}{\begin{array}{l} \mathrm{Volume of nucleic acid extracted used for}\;\mathrm{RT}-\mathrm{qPCR}\;{\left(\mathrm{ml}\right)}^{*}\times {\mathrm{Concentration factor}}^{\mathrm{ss}}\\ \; \end{array}}\times 1,000 \end{array}$$

$${*}_{\mathrm{If}\;10\;\mu l\;\mathrm{of the nucleic acid extract is used in}\;\mathrm{RT}-\mathrm{qPCR assay the value in}\;\mathrm{ml}\;\mathrm{is}\;0.01.}$$

$${\mathrm{ss}}_{\mathrm{Concentration factor}=\frac{\mathrm{Wastewater sample volume used}\;\left(\mathrm{ml}\right)}{\mathrm{Volume of nucleic acid extracted}\;\left(\mathrm{ml}\right)}}$$

Virus concentration levels (genome copies per L) were normalized by multiplying with the daily WWTP flow rate of specific WWTP and then dividing by the number of people served to get daily load/persons in sewershed [million gene copies (MGC)/person/day]. However, viral concentration levels in all samples from Mikililand WSP were only expressed as genome copies/L of RNA due to a lack of daily flow rate data.

### Estimating the number of infectious individuals

The number of daily reported COVID-19 cases in Addis Ababa during the study (February to November 2023) was obtained from the Public Health Emergency Management Center at the Ethiopian Public Health Institute. The number of residents served by the WWTP was obtained from the respective Woreda offices and the Addis Ababa Water and Sewerage Authority (AAWSA) (Table 1). Using two different approaches that have been previously published, the number of infected individuals within each WWTP’s service area was calculated (27, 46).

The equations used for calculation are indicated below:

Method 1 (Equation 1) (27):


(1) $$\begin{array}{l} \mathrm{Predicted Infected person}\\ =\frac{\left(\frac{\mathrm{RNA}\;\mathrm{copies}}{\mathrm{Liter wastewater}}\right)\times \left(\frac{\mathrm{Liter of wastewater}}{\mathrm{day}}\right)}{\left(\frac{g\;\mathrm{of feces}}{\mathrm{person}-\mathrm{day}}\right)\times \left(\frac{\mathrm{RNA}\;\mathrm{copies}}{g\;\mathrm{of feces}}\right)} \end{array}$$

A positive individual is thought to excrete 128 g of feces per person per day and shed 10^7^ RNA copies per g of feces (27).

Method 2 (Equation 2) (46):


(2) $$\begin{array}{l} \mathrm{Predicted infected person}\\ =\frac{\mathrm{Number of}\;\mathrm{RNA}\;\mathrm{copies}\;\mathrm{per}\;\mathrm{liter of wastewater}}{\mathrm{Contribution of}\;\mathrm{RNA}\;\mathrm{copies}\;\mathrm{per}\;\mathrm{person to total wastewater}} \end{array}$$

10^7^ RNA copies/g of feces was multiplied by 120 ml of the volume of feces excreted by humans (considering the density of feces as 1.07 g/ml), and total wastewater (L) received at WWTP (46).

### Statistical analysis

According to the Kolmogorov–Smirnov test, the viral concentration data were not normally distributed. We tested for significant differences in viral concentration (gc/ml) across sites using a Kruskal–Wallis rank sum and pairwise Wilcoxon tests. Spearman correlation was used to assess the correlation between reported cases and viral data. All data analysis was performed using Inter cooled STATA version 14.0 (College Station, TX, USA). The graphs are presented using Power BI.

### Ethical statement

Informed consent is not applicable for environmental wastewater samples as no human subject is involved. However, for the use of reported COVID-19 cases data from Addis Ababa, permission was granted from the Ministry of Health, which owns the data. For COVID-19 protection, care was taken during sample collection and analysis using personal protection equipment and a standardized method. All respective bodies (government and non-government) participated in this study adhered to the sample collection and laboratory testing protocols. In addition, this study obtained ethical clearance from the Ethiopian Public Health Institute Scientific and Ethical Review Office (Ref. EPHI 6.13/577). Official approval was obtained from AAWSA, the government body that administers Addis Ababa city’s water supply and sewerage services. Access to the treatment plant and site-level information was obtained from this authority.

## Results

### Method optimization for wastewater-based SARS-CoV-2 detection

SARS-CoV-2 detection and quantification from wastewater samples using the Moore swab method is a relatively new method in Ethiopia, apart from its use in polio surveillance. The comparison of the on-site autosampler method in place at KTP and the Moore swab sampling technique for SARS-CoV-2 detection in wastewater is presented in Table 2.

**Table 2:** Comparison of Moore swab sampling technique and autosampler.

Date	ORF1ab of swab	ORF1ab of auto	N gene of swab	N gene of auto	S gene of swab	S gene of auto
23 February 2023	32.001	32.543	32.719	33.390	31.571	30.163
27 February 2023	33.361	32.085	33.638	34.101	33.663	30.564
2 March 2023	32.753	32.111	36.441	33.578	31.223	31.115
6 March 2023	30.408	29.154	28.942	28.352	30.082	29.475
9 March 2023	30.848	31.617	29.348	29.414	31.593	31.449
13 March 2023	30.443	29.882	28.884	28.241	31.170	30.266
15 March 2023	31.665	29.841	30.119	28.559	31.348	29.577
16 March 2023	31.606	31.185	30.579	29.571	30.761	31.431

The Ct-value of the target genes detected using autosampler and Moore swab sampling technique. The Ct values of the target genes between autosampler and swab sampling technique were not significantly different (ORF1ab, *p* = 0.1386; N gene, *p* = 0.0858; S gene, *p* = 0.1386).

**Figure 2:**
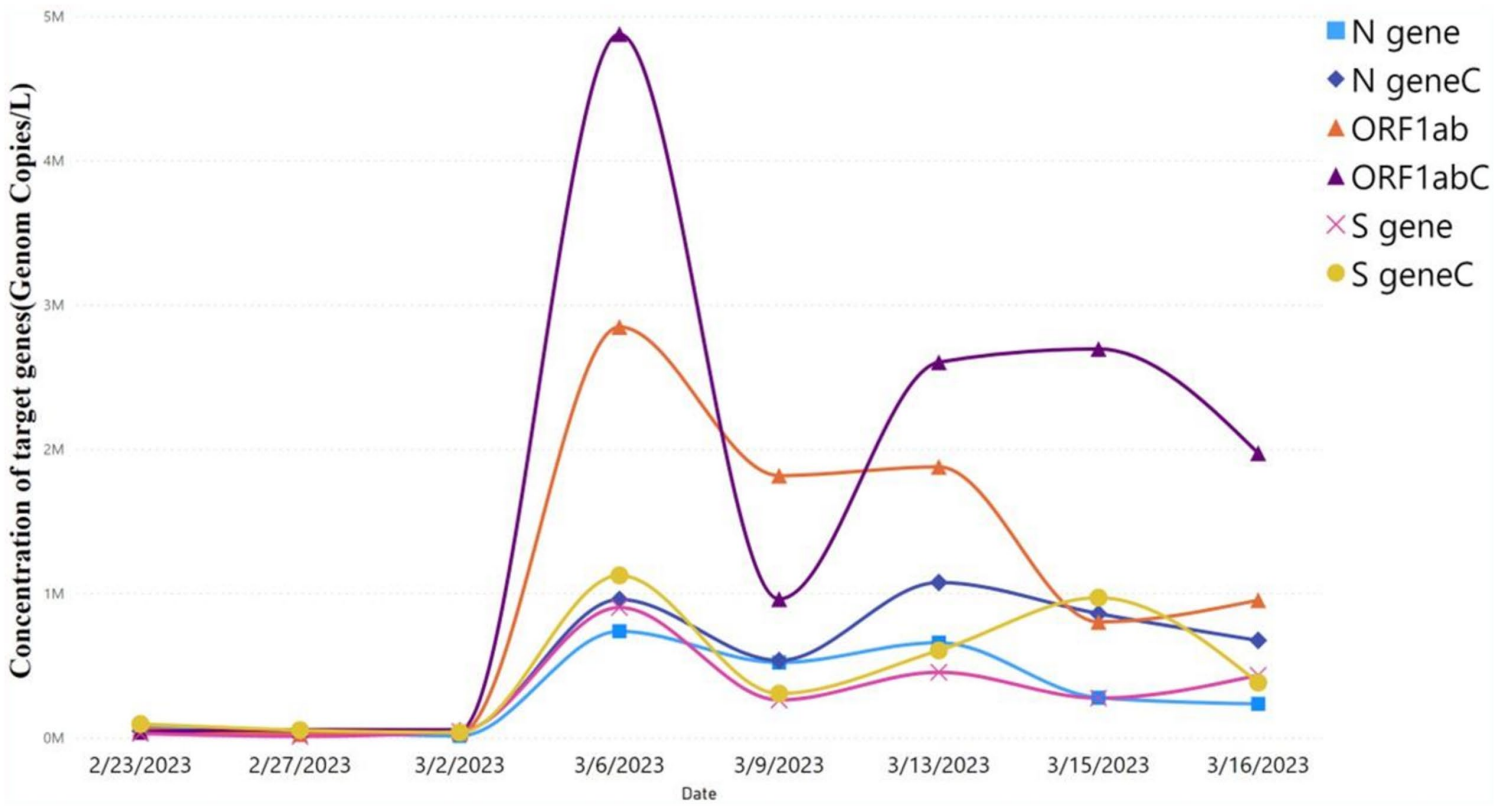
Viral concentration of autosampler and Moore swab sampling technique. Comparison of viral target genes concentration level using autosampler vs. Moore swab sampling technique. ORF1ab, N gene, and S gene were the target genes. Target genes with “C” represent the concentration of viral target genes using the autosampler, whereas target genes without “C” represent the viral target concentration using the Moore swab sampling technique. The level of concentration of the target genes predicted by the autosampler vs. swab sampling technique was not significant (*p* > 0.05).

As shown in the table, there is no significant difference in the Ct values of the target genes (ORF1ab, N, and S genes) between the autosampler and Moore swab sampling techniques. Moreover, viral concentrations (gc/L) of the target genes were not significantly different using the autosampler and Moore swab sampling technique (*p* > 0.05; Figure 2). Although the autosampler method of wastewater sampling is reliable, it has limitations that impede effective surveillance, especially from small catchments with limited accessibility. Since Moore swab sampling is more cost-effective and requires fewer resources to process, we decided to continue our monitoring of wastewater for SARS-CoV-2 using this technique.

### Wastewater-based SARS-CoV-2 qualitative test result

Wastewater samples collected from 21 February 2023 to 9 November 2023 in Addis Ababa at KTP, Bulbula WWTP, and Mikililand WSP were tested for SARS-CoV-2 by qRT-PCR. A total of 323 wastewater Moore swab samples were tested. Each run had negative controls and produced all negative results. Of these, 304 (94%) tested positive for SARS-CoV-2 by qRT-PCR, defined as a Ct value of <37 for two or more SARS-CoV-2 target genes. In addition, 14/323 (4%) of the samples tested were inconclusive for SARS-CoV-2 by qRT-PCR, defined as a Ct value of <37 for one SARS-CoV-2 target gene only in duplicate testing, and only 5/323 (2%) were negative, defined as a Ct value of ≥37 for three SARS-CoV-2 target genes and a Ct value of <32 for MS2 (internal control). Around 95% of samples from KTP were positive, whereas 2% were negative and 3% were inconclusive. Approximately 90% of the Bulbula samples were positive, with the remaining 3% negative and 7% inconclusive. Finally, 97% of the Mikililand samples were positive, 3% were inconclusive, and no negative results were found.

To determine the presence of SARS-CoV-2 RNA, 33 treated effluent water samples were taken from these three wastewater treatment plants. From each wastewater treatment plant, 11 treated wastewater samples were collected. All treated samples were collected in the morning from 8:00 a.m. to 12:00 p.m. by collecting 500–1,000 ml of water in sterile plastic containers. The collected samples were transported using ice and concentrated within 24 h, using the same process as influent wastewater. The SARS-CoV-2 extraction and detection procedure for treated wastewater samples was the same as for influent wastewater. Of the total 33 samples, 22 treated wastewater samples from Kality and Bulbula WWTP were negative, whereas two of the total treated samples from the Mikililand stabilized pond were positive. Five treated samples from the Mikililand stabilized pond were inconclusive, and the remaining four samples were negative.

### Quantification RNA of SARS-CoV-2 in wastewater

The wastewater samples that tested positive for SARS-CoV-2 RNA by qualitative methods were subjected to quantitative PCR for three targets (ORF1ab, N gene, and S gene). The performance efficiency range of the ORF1ab, N, and S genes among the test runs was 91.8 to 105.7, 93.0 to 109.8, and 88.0 to 104.6, respectively, and the detailed results are summarized in Supplementary Table S1.

**Figure 3:**
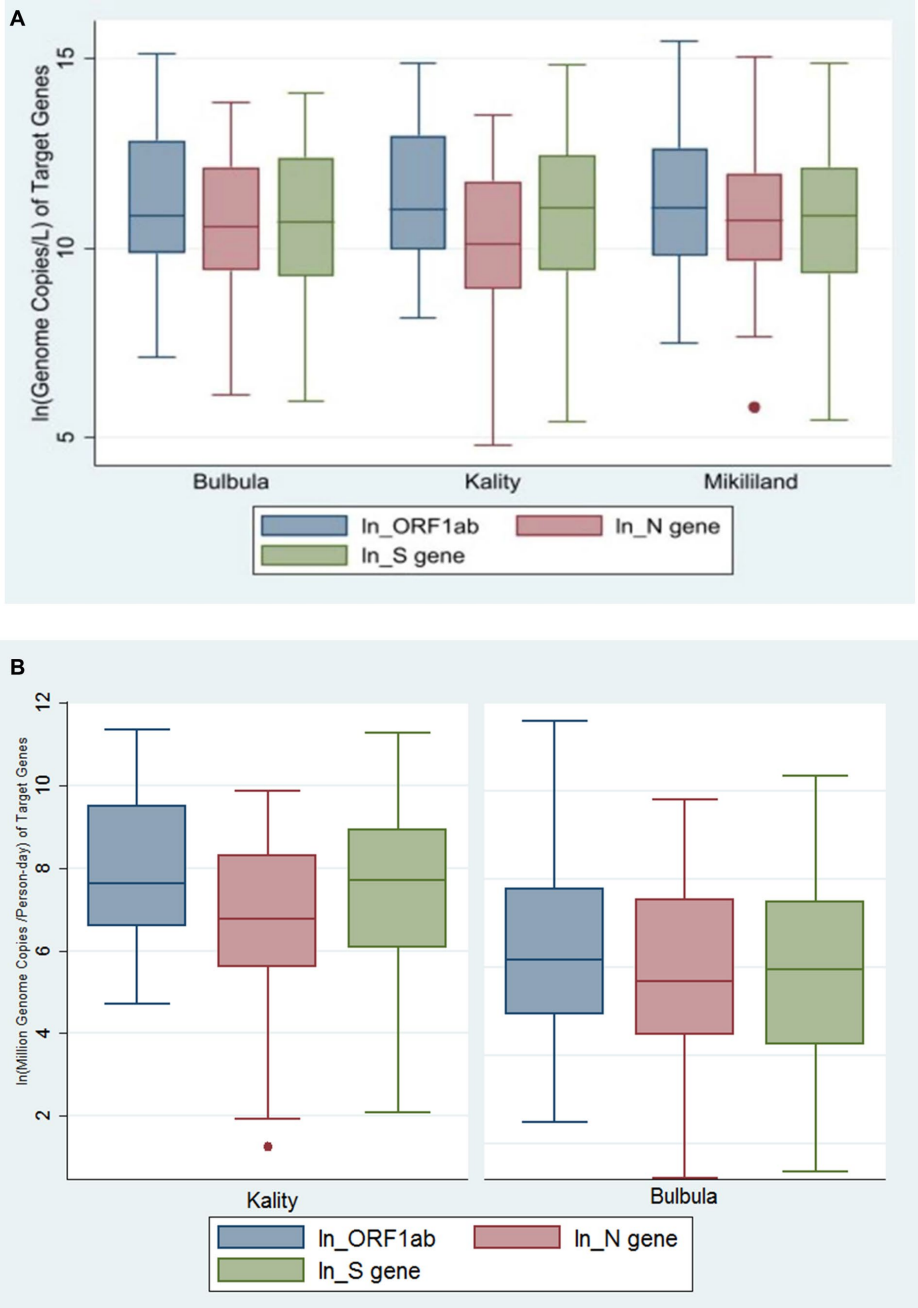
**(A)** Genome copies per L of SARS-CoV-2 gene targets in three wastewater treatment plants using Box–Whiskers plot. The data represents the average number of SARS-CoV-2 gene copies for ORF-1ab gene, N gene, and S gene per L of wastewater sample obtained in the influent wastewater samples from the Three WWTPs. **(B)** The viral concentration of daily load per person in sewershed (MGC per Person-day) of SARS-CoV-2 gene targets in two wastewater treatment plants using Box–Whiskers plot.

The concentration of these three viral targets in the influent wastewater samples across the three wastewater treatment plants (WWTPs) is presented using a Box–Whisker plot (Figure 3A). The median viral concentration (gc/L) and (interquartile range [IQR]) obtained for ORF1ab, N gene, and S gene in positive samples from KTP was 60,388 (21544–430,339), 26,355 (7,748–125,372) and 6,2,573 (12,221–24,9,039), respectively. Similarly, the median viral concentration (gc/L) and IQR for ORF1ab, N gene, and S gene from Bulbula-positive samples was 52,780 (19,078–375,512), 38,301 (12,273–186,201), and 43,549 (10476–240,648), respectively. Whereas the median viral concentration (gc/L) and IQR for ORF1ab, N gene, and S gene in Mikililand-positive samples was 64,762 (18087–309,415), 45,580 (15,681–158,475), and 51,454 (11,318–184,333), respectively. Hence, there was no significant difference among the study sites in viral concentration: ORF1ab (*p* = 0.7341), N gene (*p* = 0.2087), and S gene (*p* = 0.8721). The detailed viral load of each positive sample is presented in Supplementary Table S2_sheet 1.

After normalization of virus concentration levels (gc/L) using daily flow rate and number of people served by each WWTP, the median viral concentration of daily load per person in sewershed (million genome copies [MGC/person-day]) and IQR was generated. Accordingly, the values for ORF1ab, N gene, and S gene in positive samples from KTP were 2055 (725–13,400), 861 (268–4,016), and 2,221 (436–7,607), respectively. Whereas, for Bulbula-positive samples, the results for ORF1ab, N gene, and S gene were 477 (136–2,387), 295 (871854), and 383 (69–1786), respectively. Therefore, there was a significant difference among the study sites in viral concentration of daily load per person in sewershed: ORF1ab (*p* < 0.0001), N gene (*p* = 0.0008), and S gene (*p* < 0.0001). The viral concentration of daily load per person in the sewershed of three viral targets in KTP and Bulbula WWTPs is presented using a Box–Whisker plot (Figure 3B).

#### Trend of viral concentration in wastewater and correlational analysis against COVID-19 daily cases

**Figure 4:**
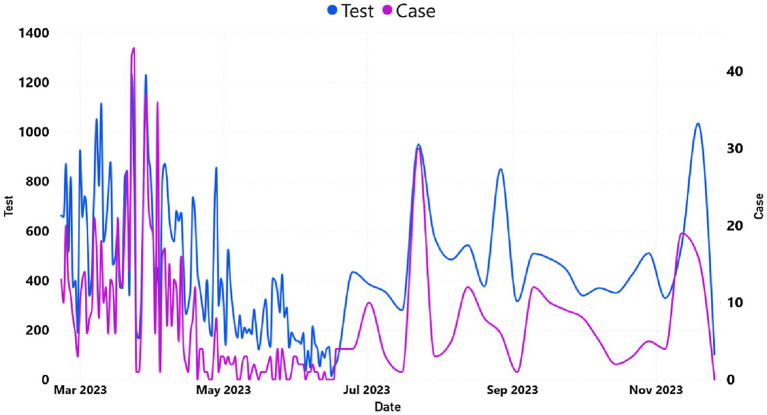
The trend of daily COVID-19 cases and tested individuals in Addis Ababa. The *y*-axis on the left represented the number of tested cases, whereas the *y*-axis on the right represented the number of reported cases.

Figure 4 demonstrates the dynamics of SARS-CoV-2 tests performed and the number of reported COVID-19 clinical cases for the year 2023. Daily reported COVID-19 cases of Addis Ababa were presented in Supplementary Table S2_sheet 2. A significant decrease of daily cases during the months of April and May 2023 presented in line with the decrease in frequency of COVID-19 testing.

**Figure 5:**
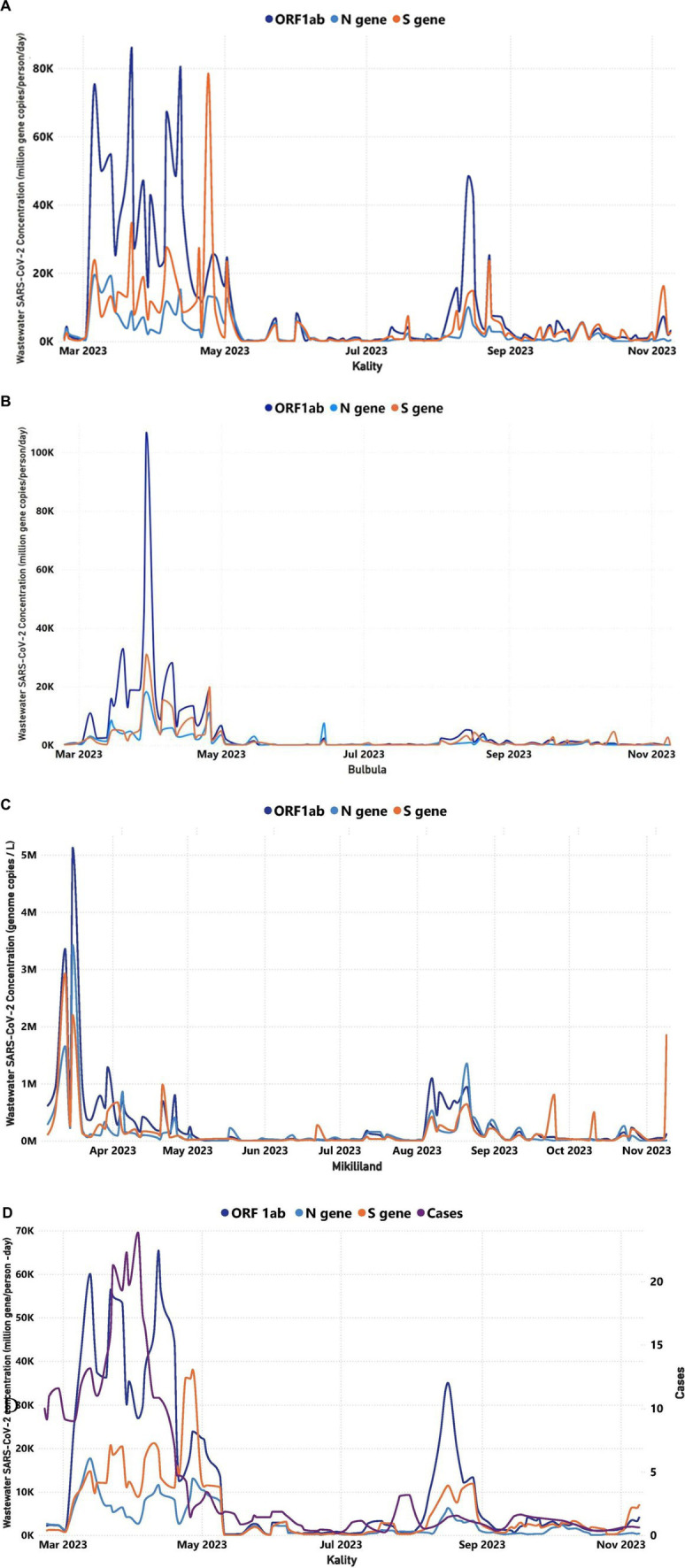
Trends in the viral target genes concentration of wastewater over time for three WWTPs in Addis Ababa (February 22, 2023–November 9, 2023): **(A)** Trends in the viral target genes concentration of wastewater over time for KTP, **(B)** Trends in the viral target genes concentration of wastewater over time for Bulbula WWTP, **(C)** Trends in the viral concentration of wastewater over time for Mikililand WSP, and **(D)** A comparison between the COVID-19 cases illustrated in purple color line that were reported in Addis Ababa and the SARS-CoV-2 target genes concentrations in KTP. The *y*-axis on the left represented the MGC/person-day of the target genes, whereas the *y*-axis on the right represented the number of reported cases. The correlation between the trend of daily reported cases and RNA concentration was significant (*p* < 0.05) in Kality. The number of cases and average SARS-CoV-2 concentration is based on the 7-day rolling average.

Positivity rates were in line with viral concentrations predicted by the three WWTPs (Figures 5A–D).

Virus concentration levels, as determined through WWTP testing, were normalized for the flow rate and number of people served. KTP is the oldest centralized system, mostly serving residents in the central, southern, and eastern parts of Addis Ababa. Figure 5A demonstrates the wastewater concentration of the target genes in samples obtained from KTP increasing sharply starting 2 March (ORF1ab = 1,123 MGC/person/day, N-gene = 1,234 MGC/person/day, and S gene = 742 MGC/person/day) to 13 March (ORF1ab = 60,066 MGC/person/day, N-gene = 17,707 MGC/person/day, and S gene = 14,773 MGC/person/day; Figure 5A). Then the wastewater concentration of the target genes fluctuated up to 27 April within the range of ORF1ab = 12,453–65,424 MGC/person/day, N-gene = 2,622–17,707 MGC/person/day, and S gene = 8,815–38,031 MGC/person/day. Then the concentration decreased sharply from 1 May up to 10 May and sustained less than 1,000 MGC/person/day of each target genes up to 5 July. Subsequently, the concentration in KTP increased by 3 October and decreased again by 24 October (Figure 5A). The trend of concentration of the target genes in wastewater samples of Bulbula and Mikililand WSP was almost similar to that of the concentration trend of KTP and with a bit of difference in time of increments or decrements (Figures 5B,C).

The case-based surveillance unit in EPHI does not have a daily active cases report for the exact residents that are served by each WWTP. However, considering the large population coverage of the KTP (i.e., serving more than one-third of the population and wide geographic coverage), it was found important to make a trend analysis of Addis Ababa daily cases against the trend of concentration for the target genes in wastewater samples collected from KTP and Bulbula WWTP.

As indicated in Figure 5D, active clinical case counts doubled from 23 February to 23 March (in 9–21 active cases). This was reflected in a 15-fold increase in the average concentration of target genes in the wastewater (ORF1ab increased by a factor of 27; the N gene increased by a factor of 7, and the S gene increased by a factor of 13). Moreover, the increase in viral target positivity in the wastewater occurred approximately 10 days ahead of the increase in reported clinical cases. Again, at a later moment in the year, a more limited increase of active case counts from 17 July to 26 July (from zero to three active cases) was preceded by a wastewater increase starting from 5 July (ORF1ab = 356 MGC/person/day, N gene = 165 MGC/person/day, and S gene = 142 MGC/person/day), 12 days earlier. This increase lasted until 16 August (ORF1ab = 33,318 MGC/person/day, N gene = 5,285 MGC/person/day, and S gene = 10,790 MGC/person/day).

The finding indicates a positive correlation between the trend of weekly average SARS-CoV-2 MGC number in wastewater samples of WWTPs and the cumulative weekly reported COVID-19 cases in Addis Ababa. These were statistically significant for all three sites: KTP (0.5648, *p* = 0.0002), Bulbula (0.4052, *p* = 0.0116), and Mikililand (0.4247, *p* = 0.0098; Supplementary Table S3).

#### Estimated numbers of COVID-19-infected individuals and correlation with reported cases in Addis Ababa

**Figure 6:**
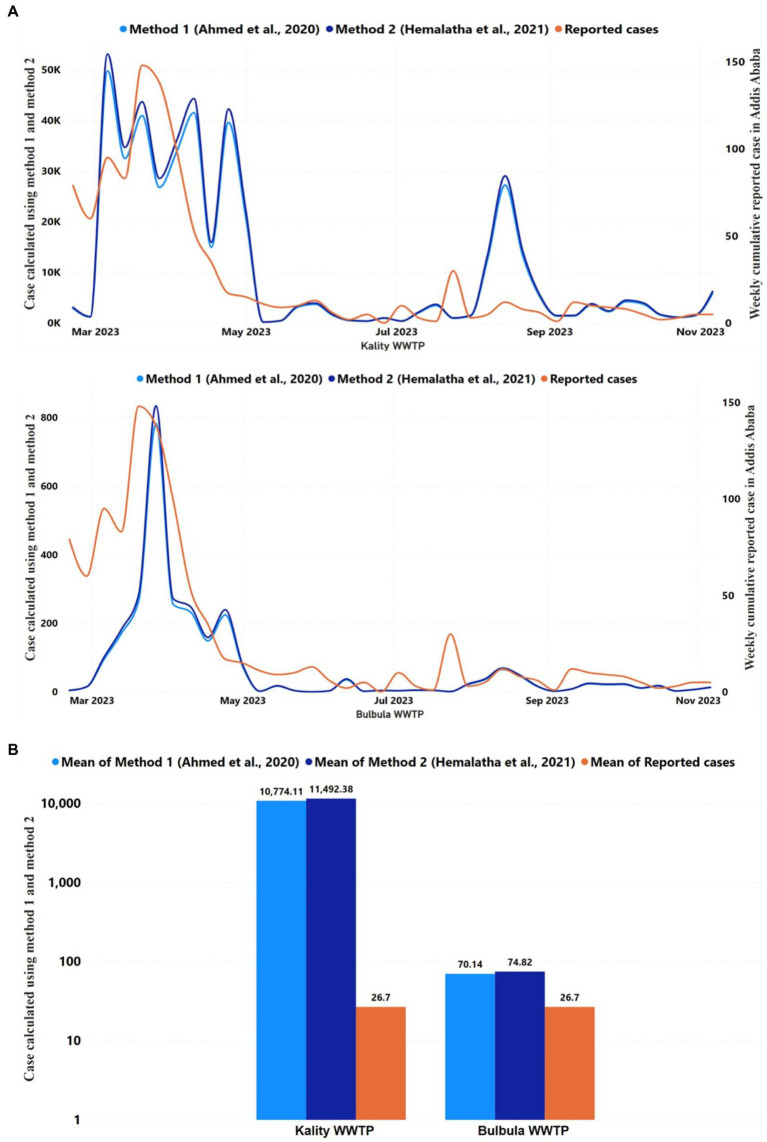
Reported and predicted COVID-19 infected cases across 2 WWTPs, **(A)** Trends in the COVID-19 reported cases and daily average predicted infected individuals using Ahmed et al. (27) and Hemalatha et al. (46) methods in Kality and Bulbula WWTP. The *y*-axis on the left represented the number of predicted cases using two methods, whereas the *y*-axis on the right represented the number of reported cases. The correlation between the trend of cumulative weekly reported and daily predicted COVID-19 cases was significant (*p* < 0.05) among the two WWTPs. **(B)** The figure represents the mean of COVID-19 reported cases of each WWTP during the study period and the predicted infected individuals using the Ahmed et al. (27) and Hemalatha et al. (46) methods for each WWTP for the study period.

Two methods were used to estimate the number of daily infected individuals among the population served by KTP and Bulbula WWTP based on the SARS-CoV-2 gene copy number obtained from the wastewater samples (27, 46). The numbers of daily predicted infected persons using method 1 and method 2 in KTP were similar and ranged from 10^2^ to 10^4^, as represented in Figure 6A. At Bulbula WWTP, these numbers were in the range of 10^0^–10^4^. The daily predicted infected individuals from KTP were 330 times the median value higher than the weekly cumulative reported COVID-19 cases (Table 3). The median predicted SARS-CoV-2 infected people of method 1 and method 2 from Kality was 3,303 and 3,523, respectively, whereas the median of weekly cumulative reported COVID-19 cases was 10. Correlational analyses of reported cases trend with the estimated number of infected individuals trend are shown in Figure 6A and Supplementary Table S3. Similarly, the two methods resulted in higher mean values of daily predicted infected individuals from WWTPs compared to weekly cumulative reported COVID-19 cases (Figure 6B). The predicted number of infected individuals using the two methods followed a decreasing trend similar to the reported COVID-19 cases in Addis Ababa, and a statistically significant correlation was observed with data from KTP WWTP using Spearman correlation (*r* = 0.5307; *p* = 0.0006) and Bulbula WWPT (*r* = 0.4816; *p* = 0.0022). However, there is a significant difference between the number of predicted cases and reported cases for each surveillance week (*p* < 0.0001 for KTP and *p* = 0.0029) for Bulbula WWPT.

**Table 3:** The median Reported COVID-19 cases and predicated infectious cases.

Name	Median COVID-19 reported cases from Addis Ababa	Median predicated COVID-19 cases	
		Method 1 (27)	Method 2 (46)
Kality WWTP	10	3,303	3,523
Bulbula WWTP	10	17	18

## Discussion

Numerous studies conducted since the beginning of the COVID-19 pandemic have shown that WBE is a useful tool for tracking the evolution of the pandemic and providing early warning signs for the emergence or reemergence of public health threats (47, 48). The SARS-CoV-2 limit of detection in wastewater is principally determined by three laboratory procedures: virus concentration, RNA extraction, and qRT-PCR. The concentration method used here is known to preferentially bind intact virus particles but not cell-free nucleic acid. Thus, using other crude concentration methods or laboratory procedures without concentration may overestimate the intact viral burden. Using a technology that binds intact virus particles also provides greater evidence of active infection vs. cleared viral nucleic acid. Grab and autosampler sampling are the two most common wastewater sampling methods, but grab sampling has drawbacks in terms of missing viral shedding discharges to sewers, and autosampler has limited accessibility (49). Our result showed that the concentration of target genes was a bit higher in the autosampler compared to the Moore swab sampler (Figure 2). The primary cause of this discrepancy may be the Moore swab or gauze sampling methods’ uptake rates, which could have been affected by inhibitors or virus losses after 8 hours of contact to the wastewater samples (50), which in the current study was installed for 24 h. However, no significant difference in the Ct value and viral concentration was observed between autosampler and Moore swab samples for SARS-CoV-2 target genes (ORF1ab, N, and S genes), which is consistent with other studies (51). We conclude that Moore swab sampling is a more economical and resource-efficient sampling technique for the monitoring of SARS-CoV-2 in wastewater in our low-resource setting and may be extended to other pathogens of interest.

In our study, SARS-CoV-2 RNA was detected in a majority of influent wastewater samples (94%). This high rate revealed a much higher COVID-19 prevalence than actually clinically detected. A prior study using an antibody prevalence analysis showed that there was a significant underreporting of COVID-19 cases in Ethiopia (52). This can be explained by the fact that the far majority of actual COVID-19 cases in Addis Ababa are either mild or asymptomatic, with patients not seeking healthcare and testing services (19).

The positive SARS-CoV-2 detection rate in Addis Ababa was approximately identical to that of Kenya (81%) using the same technique of collection and testing (53), but higher than that of Malawi (8%) using samples taken from rivers and defunct WWTPs (33). In Malawi, samples from the defunct WWTP were found to have higher SARS-CoV-2 positive rates (21%) than river water samples (7%). Thus, the discrepancy in positive rate between our findings and Malawi might be attributed to the variance in viral shedding discharges into sewer lines of WWTP, rivers, and defunct WWTP (54). Furthermore, the variation in results may be attributed to differences in flow rate, methodology, data collection, and actual virus concentration differences since Malawi used grab sampling and polyethylene glycol (PEG) with no internal control (MS2) and potentially generated false negatives (55). The intensity of community transmission of SARS-CoV-2, the timing of the study, and the population served might also be important variables that make a difference observed for the positivity rates. The SARS-CoV-2 viral copy numbers (GC/L) of the amplification target genes were similar over the year 2023 (ORF1ab =10^3^–10^6^, N and S genes =10^2^–10^6^ gc/L) in all three WWTP influent wastewater samples. This result shows the genome copies per 10 ml were not different at each treatment plant.

However, we observed a significant difference in terms of daily load per person for all target genes between KTP and Bulbula WWTPs; this is attributable to the difference in the prevalence of infected individuals that are served by each plant and the flow rate of the treatment plants.

For treated wastewater samples, the SARS-CoV-2 RNA was absent in all (*n* = 11 each) of the treated wastewater samples from two wastewater treatment plants (KTP and Bulbula). This result suggests that the UASB used in KTP and MBR technology used in Bulbula can successfully remove SARS-CoV-2 from wastewater to levels that are under the limit of detection of qPCR. However, at Mikililand WWTP (using a stabilization pond) SARS-CoV-2 RNA was still detected in 2 treated wastewater samples (*n* = 11), 5 being inconclusive. This demonstrates the limitations of the applied treatment method for viral eradication. Similar results were found in research conducted in Spain (56), where 2/18 of treated samples still tested positive for SARS-CoV-2.

In general, it is important to emphasize that the models used in this study may be crude compared to some of the more recently developed models generated. The Pepper Mild Mottle Virus (PMMoV), which is the most abundant RNA virus in human feces and occurs naturally in wastewater, has been used in recent studies (57, 58) to normalize qRT-PCR data. This approach may be more reliable in estimating the depth of infection in a community and could be used in an Ethiopian setting as well in the future.

Generally, we found fluctuating viral concentrations (MGC per person-day) over the study period. The overall change in the SARS-CoV-2 viral load in wastewater is positively correlated with reported COVID-19 clinical cases though the clinical testing frequency was low.

Thus, our result shows a significant positive correlation between trend viral loads in wastewater and reported COVID-19 clinical cases. This finding is consistent with previous studies in New York (59), India (60), and Hong Kong (61). In our setting, the increase in viral concentration started in the wastewater approximately 7–14 days ahead of the increase in COVID-19 clinical cases, as reported elsewhere (21). Furthermore, the amount of virus in wastewater did not drop off when the number of COVID-19 clinical cases significantly declined, which is consistent with a prior study that showed viral RNA might remain in fecal samples for up to 10 days (62).

The higher daily predicted infected persons from KTP, which was 330 times greater than the weekly cumulative recorded COVID-19 cases, revealed the high prevalence of asymptomatic individuals shedding SARS-CoV-2 to the sewage system in the catchment area. This is in line with previous studies in Ethiopia that have shown a significant inverse correlation between parasite infection prevalence and lack of COVID-19 symptoms due to shifts in activation status of the immune system (63, 64). Most people infected with SARS-CoV-2 in Ethiopia do not get sick (15), partly due to widespread parasitic infections (65, 66) and they may not seek medical care. Alternatively, the difference may be a result of the delay in active case reporting because qRT-PCR testing is biased as many tested individuals are not randomly undergoing diagnostic procedures, but their participation is motivated by the onset of symptoms either in themselves or in the person sharing their work or living environment, the prevalence of asymptomatic infection within the community as measured by rapid antigen tests might be underestimated due to sensitivity issues (67). On the other hand, the daily predicted infected individuals from Bulbula WWTP were merely 1.8 times the median value of the weekly cumulative reported cases in Addis Ababa (Table 3). The difference in predicted infected people in KTP and Bulbula is primarily attributable to the difference in flow rate at the treatment plants (Supplementary Table S3), which may further depend on the number of people served. Accordingly, the more the population served, the more viral shading is in the wastewater.

## Conclusion

In conclusion, this study was undertaken to assess the presence of SARS-CoV-2 in the wastewater samples in three WWTPs in Addis Ababa and evaluate its predictive value for clinical COVID-19 case reporting. Nanotrap® Microbiome A particles, Nanotrap® Enhancement Reagent 2 method, and Moore swab collection methods appeared to be effective in concentrating the virus from wastewater and can, therefore, be used in resource-limited settings. The significantly higher rate of SARS-CoV-2 detection from wastewater samples suggests a hidden high prevalence of COVID-19 disease in the population that remains overtly asymptomatic and/or underreported. Effluent wastewater treatment was only partly successful in making SARS-CoV-2 RNA undetectable at the KTP and Bulbula WWTP but not at Mikililand, indicating cautiousness is recommended. The peak in SARS-CoV-2 positivity rates in wastewater typically indicated a rise in clinical COVID-19 cases within 1–2 weeks later. The wastewater surveillance experience developed through this project can be applied to other national priority diseases in the future.

## Data Availability

The datasets presented in this study can be found in online repositories. The names of the repository/repositories and accession number(s) can be found in the article/Supplementary material.
