# Supplementary material for: Longitudinal wastewater-based surveillance of SARS-CoV-2 during 2023 in Ethiopia
Source: Front Public Health. 2024 Oct 7;12:1394798. doi: 10.3389/fpubh.2024.1394798 (PMC11491403; doi:10.3389/fpubh.2024.1394798)
Supplement: Supplementary file 1 [file Table_1.DOCX]

# **Title**: Longitudinal Wastewater - Based Surveillance of SARS-CoV-2 in Addis Ababa, Ethiopia

**Table S1**: Quality performance of qRT-PCR assay for SARSCoV-2 gene targets

| Efficiency | | | Slope | | | R2 | | | Y-Intercept | | |
| --- | --- | --- | --- | --- | --- | --- | --- | --- | --- | --- | --- |
| ORF 1ab | N gene | S gene | ORF 1ab | N gene | S gene | ORF 1ab | N gene | S gene | ORF 1ab | N gene | S gene |
| 98.024 | 92.950 | 88.001 | -3.370 | -3.503 | -3.648 | 0.967 | 0.986 | 0.965 | 34.884 | 36.080 | 33.525 |
| 98.109 | 101.815 | 97.928 | -3.368 | -3.279 | -3.373 | 0.975 | 0.992 | 0.982 | 38.343 | 35.165 | 36.516 |
| 98.387 | 104.610 | 103.917 | -3.361 | -3.216 | -3.231 | 0.990 | 0.987 | 0.994 | 32.829 | 32.603 | 34.804 |
| 95.006 | 97.157 | 100.703 | -3.448 | -3.392 | -3.305 | 0.999 | 0.999 | 0.981 | 35.585 | 33.590 | 32.996 |
| 91.820 | 97.172 | 100.298 | -3.535 | -3.392 | -3.315 | 0.994 | 0.995 | 0.984 | 34.386 | 34.267 | 33.420 |
| 98.109 | 101.815 | 97.928 | -3.368 | -3.279 | -3.373 | 0.975 | 0.992 | 0.982 | 38.343 | 35.165 | 36.516 |
| 105.728 | 109.783 | 112.655 | -3.192 | -3.108 | -3.052 | 0.990 | 0.987 | 0.992 | 35.430 | 33.576 | 35.889 |
| 94.679 | 99.507 | 104.048 | -3.456 | -3.334 | -3.229 | 0.997 | 0.997 | 0.980 | 36.077 | 35.629 | 35.831 |
| 96.010 | 100.408 | 105.320 | -3.421 | -3.312 | -3.201 | 0.994 | 0.997 | 0.995 | 35.725 | 33.363 | 34.502 |

The performance of standard controls (efficiency, Slope, R2 and Y-intercept) in each nine runs. The recommended values for these standard controls (efficiency, Slope, and R2) are 90 _110, >0.98, -3.1 to -3.6 respectively.

**Table S3**: Estimation of infectious persons and reported cases

| **WWTP** | **Week** | **Average flow rate (m3 per day)** | **population served by WWTP** | **weekly Average reported COVID-19 (Addis Ababa)** | **AverageSARS-CoV-2 GC/L** | **Method 1 (Ahmed et al., 2020)** | **Method 2 (Hemalatha et al., 2021)** |
| --- | --- | --- | --- | --- | --- | --- | --- |
| Akaki Kality* | week_1 | 57090 | 2000000 | 79 | 65197.4 | 2908 | 3102 |
|  | week_2 | 54744 |  | 60 | 27980.1 | 1197 | 1276 |
|  | week_3 | 53976 |  | 95 | 1180534.5 | 49782 | 53100 |
|  | week_4 | 62760 |  | 83 | 662513.1 | 32484 | 34649 |
|  | week_5 | 61112 |  | 148 | 857728.5 | 40951 | 43681 |
|  | week_6 | 58664 |  | 138 | 583901 | 26761 | 28545 |
|  | week_7 | 52280 |  | 99 | 827999.6 | 33819 | 36073 |
|  | week_8 | 57008 |  | 53 | 932705.9 | 41540 | 44310 |
|  | week_9 | 58264 |  | 35 | 328626.2 | 14959 | 15956 |
|  | week_10 | 57400 |  | 17 | 883069.3 | 39600 | 42240 |
|  | week_11 | 60384 |  | 15 | 441251.5 | 20816 | 22204 |
|  | week_12 | 70602 |  | 11 | 3556.5 | 196 | 209 |
|  | week_13 | 60280 |  | 9 | 9292.9 | 438 | 467 |
|  | week_14 | 65544 |  | 10 | 61328.3 | 3140 | 3350 |
|  | week_15 | 65352 |  | 13 | 72889.1 | 3721 | 3970 |
|  | week_16 | 51104 |  | 6 | 40026.1 | 1598 | 1705 |
|  | week_17 | 52984 |  | 2 | 12172.2 | 504 | 537 |
|  | week_18 | 67080 |  | 5 | 7123.7 | 373 | 398 |
|  | week_19 | 68240 |  | 0 | 18068.2 | 963 | 1027 |
|  | week_20 | 74352 |  | 10 | 6459.2 | 375 | 400 |
|  | week_21 | 73568 |  | 3 | 35537.1 | 2042 | 2179 |
|  | week_22 | 71072 |  | 1 | 62409.6 | 3465 | 3696 |
|  | week_23 | 65960 |  | 30 | 18555 | 956 | 1020 |
|  | week_24 | 68640 |  | 3 | 26327.8 | 1412 | 1506 |
|  | week_25 | 66920 |  | 5 | 245196.1 | 12819 | 13674 |
|  | week_26 | 70072 |  | 12 | 497641.5 | 27243 | 29059 |
|  | week_27 | 62368 |  | 8 | 277270.4 | 13510 | 14411 |
|  | week_28 | 65696 |  | 6 | 101916.7 | 5231 | 5580 |
|  | week_29 | 69768 |  | 1 | 25508.5 | 1390 | 1483 |
|  | week_30 | 69344 |  | 12 | 26161.6 | 1417 | 1512 |
|  | week_31 | 67832 |  | 10 | 67608.4 | 3583 | 3822 |
|  | week_32 | 69720 |  | 9 | 41243.5 | 2246 | 2396 |
|  | week_33 | 72344 |  | 8 | 74917.6 | 4234 | 4517 |
|  | week_34 | 70360 |  | 5 | 67916.6 | 3733 | 3982 |
|  | week_35 | 69728 |  | 2 | 28756.4 | 1567 | 1671 |
|  | week_36 | 76360 |  | 3 | 18587.6 | 1109 | 1183 |
|  | week_37 | 74736 |  | 5 | 25246.4 | 1474 | 1572 |
|  | week_38 | 80416 |  | 5 | 93245.2 | 5858 | 6249 |
| Bulbula* | week_1 | 446 | 34000 | 79 | 12518.1 | 4 | 5 |
|  | week_2 | 524.25 |  | 60 | 35535.7 | 15 | 15 |
|  | week_3 | 459.5 |  | 95 | 244059.4 | 95 | 101 |
|  | week_4 | 388 |  | 83 | 523255.8 | 171 | 182 |
|  | week_5 | 340.5 |  | 148 | 995824 | 266 | 283 |
|  | week_6 | 635.33 |  | 138 | 1417802.5 | 782 | 834 |
|  | week_7 | 501.67 |  | 99 | 646010.5 | 255 | 271 |
|  | week_8 | 507 |  | 53 | 572886.5 | 233 | 248 |
|  | week_9 | 989.33 |  | 35 | 227818.9 | 149 | 159 |
|  | week_10 | 435 |  | 17 | 618145.5 | 225 | 240 |
|  | week_11 | 214.07 |  | 15 | 417917.7 | 75 | 80 |
|  | week_12 | 361.67 |  | 11 | 7865.4 | 2 | 2 |
|  | week_13 | 505.67 |  | 9 | 56469.8 | 17 | 18 |
|  | week_14 | 315.33 |  | 10 | 14050.1 | 4 | 4 |
|  | week_15 | 123 |  | 13 | 3921.3 | 1 | 1 |
|  | week_16 | 196.67 |  | 6 | 23213.6 | 3 | 3 |
|  | week_17 | 196.5 |  | 2 | 187520.4 | 36 | 38 |
|  | week_18 | 164.67 |  | 5 | 16534.7 | 2 | 2 |
|  | week_19 | 186 |  | 0 | 28697.3 | 4 | 4 |
|  | week_20 | 233.33 |  | 10 | 19404.4 | 4 | 4 |
|  | week_21 | 171.33 |  | 3 | 38021.1 | 5 | 5 |
|  | week_22 | 167 |  | 1 | 34569.2 | 4 | 5 |
|  | week_23 | 204.33 |  | 30 | 8281.2 | 1 | 1 |
|  | week_24 | 166.33 |  | 3 | 156791.1 | 22 | 23 |
|  | week_25 | 161.33 |  | 5 | 287652.6 | 36 | 38 |
|  | week_26 | 192.67 |  | 12 | 435357.9 | 65 | 70 |
|  | week_27 | 213 |  | 8 | 276085.9 | 46 | 49 |
|  | week_28 | 156 |  | 6 | 133268.9 | 16 | 17 |
|  | week_29 | 119 |  | 1 | 27343.4 | 2 | 3 |
|  | week_30 | 304.33 |  | 12 | 56765.8 | 9 | 9 |
|  | week_31 | 257.67 |  | 10 | 105042.6 | 24 | 25 |
|  | week_32 | 372.67 |  | 9 | 73407.4 | 21 | 23 |
|  | week_33 | 384.67 |  | 8 | 70733.7 | 22 | 23 |
|  | week_34 | 414 |  | 5 | 33441.6 | 11 | 12 |
|  | week_35 | 568.33 |  | 2 | 35321.4 | 17 | 19 |
|  | week_36 | 379 |  | 3 | 9536.3 | 3 | 3 |
|  | week_37 | 291.67 |  | 5 | 29028.7 | 7 | 7 |
|  | week_38 | 300.67 |  | 5 | 55401.8 | 13 | 14 |

The correlation of predicted infected persons using two methods and reported COVID-19 cases. *Significant association between averages COVID-19 reported clinical cases versus SARS-CoV- 2 GC/L and predicted infected persons (*p* < 0.05)
